# Case Report: A case of Poirier–Bienvenu neurodevelopmental syndrome manifesting primarily as eyelid myoclonia

**DOI:** 10.3389/fped.2025.1583346

**Published:** 2025-09-03

**Authors:** Yuanyuan He, Qingqing Deng, Chen Chen, Zhanli Liu, Lingwei Weng

**Affiliations:** Department of Neurology, Hangzhou Children’s Hospital, Hangzhou, China

**Keywords:** POBINDS, eyelid myoclonia, CSNK2B, Jeavons syndrome, CK2β

## Abstract

Variants in the *CSNK2B* gene are known to cause Poirier–Bienvenu neurodevelopmental syndrome (POBINDS). Since its first report in 2017, nearly 100 cases have been documented. Epileptic seizures and intellectual disabilities are core symptoms of POBINDS. While the *CSNK2B* genotype and phenotype exhibit increasing diversity, the genotype-phenotype correlation remains unclear. In this study, we identified a novel *CSNK2B* heterozygous mutation NM_001320.7:c.268A > C (p.Thr90Pro) in a child with Jeavons syndrome, classified as a likely pathogenic under ACMG guidelines. Computational analyses predicted that the change of c.268A > C (p. Thr90Pro) might have an impact on the stability of the protein. This pathogenic mutation enriches the spectrum of *CSNK2B* gene mutations and suggests that *CSNK2B* may be a causative gene for Jeavons syndrome.

## 1 Introduction

Poirier–Bienvenu neurodevelopmental syndrome (POBINDS; OMIM 618732) is a rare autosomal dominant disorder characterized by early onset epilepsy, language impairment, varying degrees of intellectual disability, developmental delay, and autism. It was first reported by Poirier et al. in 2017 and is caused by variants in the *CSNK2B* gene (1). *CSNK2B* is located on chromosome 6p21.33, consists of 7 exons, and spans 3988 base pairs. It encodes a CK2 regulatory subunit (β, CK2β) that contains 215 amino acids. This subunit includes multiple domains such as the KET box-like domain, disruptive domain, Asp/Glu acidic domain, and zinc finger motif, making it an essential component of casein kinase (CK2). Recent studies have demonstrated that CK2 activity is negatively regulated via proteasome-mediated degradation of CK2β, while increased CK2β expression can enhance CK2 activity (2, 3). CK2 is a widely expressed serine/threonine kinase complex composed of two catalytic (CK2α/α′) and two regulatory (CK2β) subunits that form heterotetramers in three configurations: α2β2, αα′β2, or α′2β2. This complex phosphorylates hundreds of substrates and regulates various signaling pathways, including the Wnt signaling pathway, playing a critical role in cell proliferation, differentiation, apoptosis, and DNA repair (4–7). CK2β is essential for central nervous system development. Its knockout in mice results in post-implantation lethality, whereas conditional *CSNK2B* knockout impairs the proliferation and differentiation of embryonic neural stem cells in the telencephalon. Additionally, *CSNK2B* knockout in mouse embryonic stem cells leads to altered neuronal morphology, reduced dendritic number and length, and synaptic transmission defects (8, 9).

Epileptic seizures and intellectual disability are core symptoms of POBINDS. Besides craniofacial abnormalities, dysmorphic facial features and growth anomalies are common phenotypes. Some patients also present with vascular, lymphatic, skeletal, ectodermal, and other abnormalities (10, 11). In this study, we identified a novel CSNK2B mutation in a pediatric patient presenting with Jeavons syndrome features. Through computational structural analysis and ACMG-based variant interpretation, we characterized the mutation's potential impact on CK2β conformation within the topoisomerase II interaction domain, expanding the phenotype spectrum of CSNK2B-related disorders, and the first reported association between POBINDS and Jeavons syndrome.

## 2 Materials and methods

### 2.1 Patient

The patient was admitted to the Neurology Department of Hangzhou Children's Hospital in August 2023. A multidisciplinary team comprising specialists in neurology, electrophysiology, and pediatric health evaluated the patient's clinical manifestations and disease episodes. Electroclinical and radiological data, neuroimaging, cognitive and behavioral tests, morphological abnormalities, and anthropometric data were collected and assessed. The study was conducted with informed consent obtained from the patient's parents and was approved by the Ethics Committee of Hangzhou Children's Hospital.

### 2.2 Sample collection and peripheral blood genomic DNA

Following informed consent, 5 ml of venous blood was drawn from the patient using an EDTA anticoagulant tube. Genomic DNA was extracted using the MagPure Buffy Coat DNA Midi KF kit, following the standard protocol.

### 2.3 Whole exome sequencing, sequence analysis, and functional prediction

The extracted genomic DNA was fragmented into 200–250 bp segments using the Covaris LE220 ultrasonicator (Massachusetts, USA). The resulting fragments were purified, end-repaired, A-tailed, and ligated to adapters to construct a sequencing library. The target libraries were hybrid-captured using the SureSelect Human All Exon V8 capture chip (Agilent, USA), followed by library amplification. Quality control was conducted using an Agilent 2,100 Bioanalyzer and ABI StepOne. Finally, high-throughput sequencing was performed using the DNBSEQ-T7 sequencer (BGI, China).

Raw sequencing data were quality-checked using AfterQC, and low-quality or adapter-contaminated reads were removed. Filtered reads were aligned to the human hg19 reference genome using the Burrows–Wheeler Aligner software to evaluate capture efficiency. Single nucleotide variants (SNVs) and insertions and deletions (indels) were identified using the Genome Analysis Toolkit and filtered against population databases, including 1,000 Genomes, Genome Aggregation Database (gnomAD), and Exome Aggregation Consortium (ExAC). The pathogenicity of missense and splice-site mutations was predicted using the dbNSFP database, and the reported mutations were screened against the Human Gene Mutation Database and ClinVar. All mutation sites were classified according to the American College of Medical Genetics and Genomics (ACMG) guidelines for variant interpretation (12).

Differences in secondary structure between mutant and wild-type (WT) proteins were analyzed using the Self-Optimized Prediction Method with Alignment (SOPMA) database (https://npsa-prabi.ibcp.fr/cgi-bin/npsa_automat.pl?page=/NPSA/npsa_sopma_f.html). The WT protein model (AF-P67870-F1-v4) was retrieved from the AlphaFold database (https://alphafold.com/) (13), and site-directed mutagenesis and structural comparison of the pre- and post-mutation protein models were performed using the PyMOL visualization software (https://pymol.org/). Surface electrostatic potentials of the CNSK2B protein before and after mutation were analyzed and visualized using the Adaptive Poisson-Boltzmann Solver (APBS) plugin in ChimeraX software (https://www.cgl.ucsf.edu/chimerax/) (14). Finally, mutation-induced changes in protein stability were predicted using three computational tools: DUET, SAAFEC-SEQ, and DynaMut2.

### 2.4 Sanger sequencing for mutation validation

The identified potential mutation sites were validated by Sanger sequencing. PCR amplification primers were designed using Primer 3 (http://primer3.ut.ee/) and synthesized by Wuhan Yingjun Biological Engineering Technology Services. The PCR reaction mixture contained 25 µl of 2× GC buffer II, 8 µl of dNTPs, 1 µl of LA Taq enzyme, 2 µl each of forward and reverse primers (10 µmol/L), 1 µl of genomic DNA solution, and 11 µl of distilled water. All reagents, except primers, were procured from Takara Bio (Dalian, China). The PCR conditions were as follows: Pre-denaturation at 94 ℃ for 5 min; 35 cycles of denaturation at 94 ℃ for 30 s; annealing at 60 ℃ for 60 s; extension at 72 ℃ for 120 s; and final extension at 72 ℃ for 10 min. All reactions were performed using an ABI Gradient PCR machine. The amplified products were sent to Wuhan Yingjun Biological Engineering Technology Services for purification and sequencing. Sequencing results were analyzed using Chromas software.

### 2.5 Copy number variation sequencing (CNVseq)

The genomic DNA library was constructed as described above and sequenced on a DNBSEQ-T7 platform (BGI, China). Raw sequencing data were quality-checked, and low-quality or adapter-contaminated reads were removed. High-quality reads were aligned to the hg19 genome sequence using the Short Oligonucleotide Analysis Package (SOAP; Beijing Institute of Genomics). PCR duplicates were removed, the observed regions were divided based on the alignment results, and read counts within each observed region were calculated. The data were normalized to reflect fluctuations in sequencing depth, and GC content correction was applied. Candidate CNVs were filtered based on predefined thresholds to obtain final CNV results.

## 3 Results

### 3.1 Clinical case report

The patient was a 5-year-and-10-month-old male. His parents were non-consanguineous with no known genetic conditions or family history of hereditary or metabolic disorders. He was a full-term G1P1 infant delivered via cesarean section, with a birth weight of 3.65 kg. The patient had no history of hypoxia or asphyxia. Motor development was normal, and height and weight were within the standard ranges. No distinctive facial features, limb deformities, or organ malformations were observed. However, the patient exhibited delayed language development, poor cognitive ability, inattention, and hyperactivity. An intelligence assessment using the WPPSI-IV yielded the following scores: Full-scale IQ, 75; verbal comprehension index, 69; visual spatial index, 75; fluid reasoning index, 79; working memory index, 79; and processing speed index, 83.

The first epileptic seizure occurred at 46 months of age. The primary seizure type was eyelid myoclonia with absence, which was characterized by the sudden cessation of ongoing activities, eyelid twitching, and unresponsiveness. These episodes lasted from a few to tens of seconds and occurred several times daily. Occasionally, seizures were accompanied by fumbling movements of the upper limb. During the interictal period, the child exhibited normal mental status with no limb movement impairment.

At 50 months of age, the child was diagnosed with “epilepsy” at a local hospital and treated with an adequate dose of “Depakine” syrup for 20 months. However, the seizures were uncontrolled. After adding clobazam as an adjunctive antiepileptic treatment, the child has remained seizure-free for the past year, with significant improvements observed on electroencephalography (EEG).

Comprehensive laboratory and auxiliary examinations, including complete blood count, liver and kidney function tests, blood ammonia, trace element analysis, thyroid function tests, and vitamin D levels, yielded normal results. Tandem mass spectrometry of the blood and organic acid analysis of the urine revealed no abnormalities. Abdominal ultrasound and electrocardiogram were also normal.

**Figure 1:**
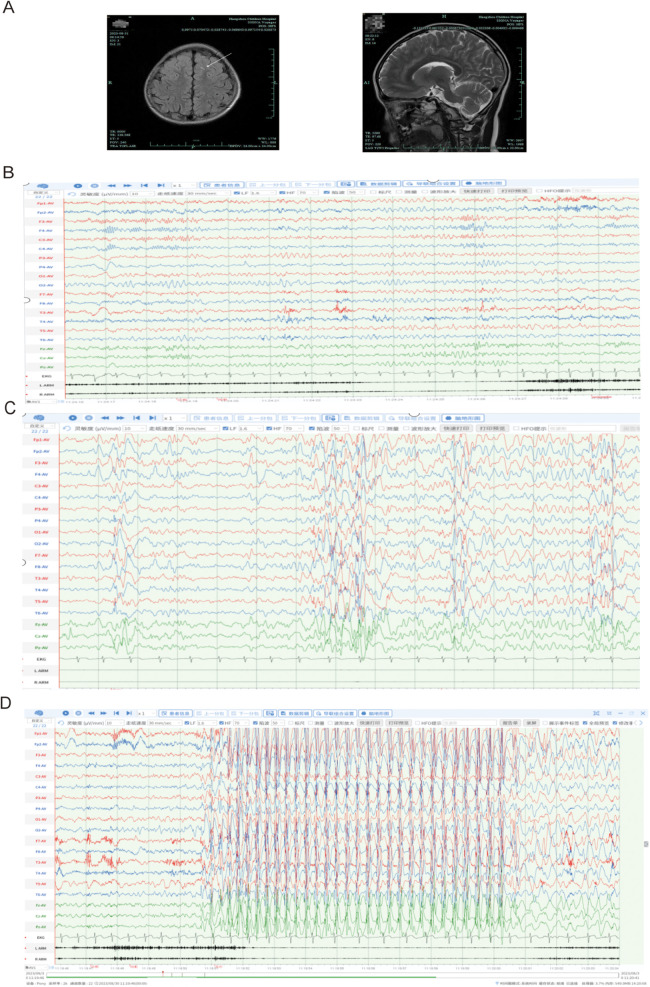
Neuroimaging and electrophysiological results. **(A)** Axial T2-weighted MRI demonstrating symmetrical frontal lobes with mild hyperintensity, suggestive of either physiologically delayed myelination or non-specific benign changes. **(B–D)** Video-EEG monitoring (international 10–20 system; average reference; LARM/RARM: bilateral upper eyelid EMG electrodes): **(B)** Wakefulness: Eye closure induces 8–8.5 Hz alpha rhythm (posterior dominance). **(C)** Sleep stage: Frequent sharp-slow wave complexes (maximal frontal) and sporadic spike-wave discharges. **(D)** Eye closure triggers 1. eyelid myoclonia (EMG onset, 1s), 2. generalized 3 Hz spike-wave activity (frontal predominance), and 3. behavioral arrest consistent with typical absence seizures (9 s duration). Supplementary Videos S1, S2 provide additional documentation of eyelid myoclonic seizures and analogous ictal events.

Cranial magnetic resonance imaging (MRI; Figure 1A) revealed punctate FLAIR hyperintensities in the local white matter of both frontal lobes. Video EEG monitoring (Figures 1B–D) detected multifocal epileptiform activity, which was more prominent during sleep, and identified eyelid myoclonia with or without absence seizures induced by eye closure during wakefulness.

### 3.2 Genetic findings

**Figure 2:**
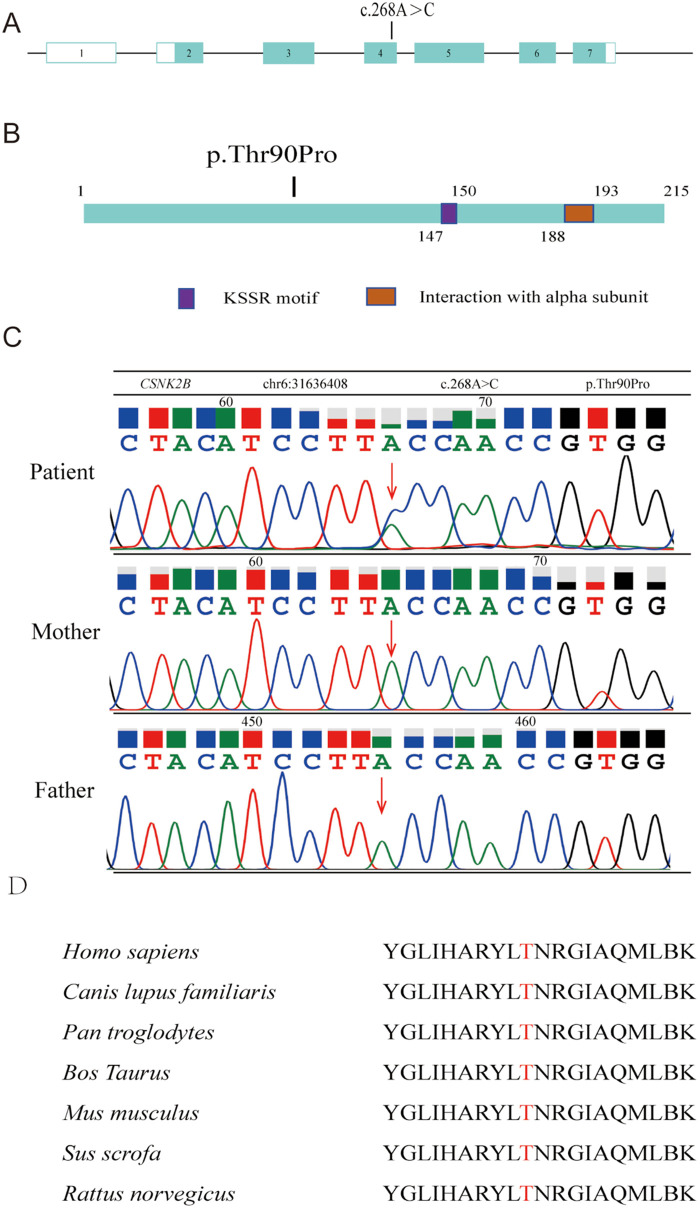
Structural diagram of the CSNK2B mutation site. **(A)** Genome structure of human *CSNK2B* with seven exons (displayed in boxes) and a mutation located in exon 4. **(B)** Schematic representation of the 215 amino acid-long CK2β protein and Mut variants. **(C)** Sanger sequencing map for the child and both parents. **(D)** Cross-species comparison revealing evolutionary conservation of Thr90 in CK2β.

Chromosomal CNV analysis revealed no abnormalities. Whole-exome sequencing revealed a missense mutation in exon 4 of *CSNK2B* (NM_001320.7: c.268A > C; p.Thr90Pro) (Figures 2A,B). According to ACMG guidelines, this variant was classified as a likely pathogenic variant based on supporting evidence from the PM2_Supporting, PM6, PP2, and PP3_Strong criteria. Sanger sequencing confirmed the heterozygous variant, which was absent in both parents (Figure 2C). This variant has not been reported in the 1,000 Genomes, ExAC, or gnomAD databases.

SIFT analysis predicted a negative effect of mutation on protein function, while Polyphen-2 predicted it as “probably damaging.” The variant received a BayesDel_noAF score of 0.487074, a REVEL score of 0.962, and a VEST4 score of 0.833. Comparative analysis of CK2β protein homologs encoded by *CSNK2B* revealed that threonine at position 90 was evolutionarily conserved across all examined species (Figure 2D).

**Figure 3:**
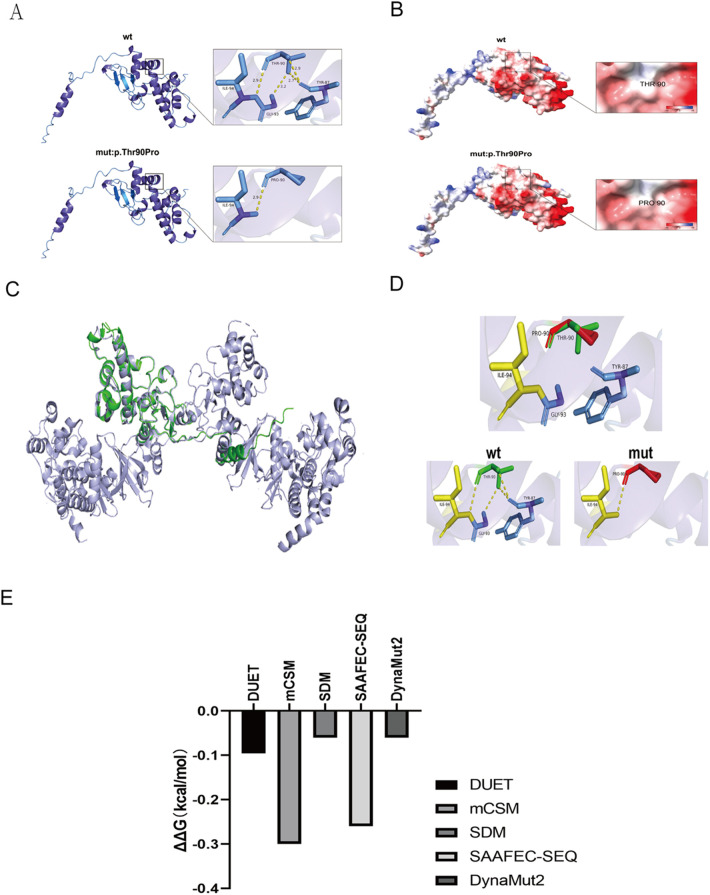
Computational analysis of mutant proteins: structural alignment, electrostatic analysis, and stability prediction. **(A,B)** Structural consequences of amino acid substitutions analyzed using AlphaFold modeling and PyMOL visualization. **(A)** Superimposed backbone/side-chain conformations of WR and mutant residues. **(B)** Comparative analysis of mutation-induced changes in the electrostatic surface potential. **(C,D)** Structural comparison of the WT and mutant CSNK2B models. **(C)** WT model (green, PDB: AF-P67870-F1 from AlphaFold DB) and mutant model (lilac) after structural alignment, demonstrating preserved core architecture. **(D)** WT (green), mutant (red), and overlapping regions (yellow), highlighting the conformational divergence. **(E)** Computational prediction of p.Thr90Pro-induced destabilization using consensus tools. DynaMut2: ΔΔG = –0.X kcal/mol (neutral/mild destabilization). DUET (mCSM/SDM consensus): ΔΔG = –0.X kcal/mol (neutral trend with mild destabilization tendency). mCSM: ΔΔG = –0.X (neutral/weak effect). SDM: ΔΔG = –0.X (neutral/mild effect). SAAFEC-SEQ: ΔΔG = –0.X kcal/mol (mild destabilization, |ΔΔG| < 0.5 suggests experimentally undetectable impact). Interpretation thresholds: DynaMut2: ΔΔG > 0 (stabilizing), −0.5–0 (neutral/mild), <–0.5 (destabilizing). mCSM: >+0.5 (stabilizing), −0.5 to +0.5 (neutral), <–0.5 (destabilizing). SDM: >+1.0 (highly stabilizing), −1.0 to +1.0 (neutral), <–1.0 (destabilizing). SAAFEC-SEQ: |ΔΔG| < 0.5 (weak), 0.5–1.0 (moderate), ≥1.0 (severe).

Analysis using the SOPMA database indicated that the p.Thr90Pro mutation alters the secondary structure of the CK2β protein, affecting alpha helices (Hh), extended strands (Ee), beta turns (Tt), and random coils (Cc) (Supplementary Materials 1, 2). AlphaFold modeling and PyMOL visualization revealed the impact of the p.Thr90Pro mutation on the three-dimensional structure of CK2β protein (Figures 3A–D). APBS analysis in ChimeraX revealed that the electrostatic potential on the protein surface remained within a neutral to negative range before and after the mutation, suggesting that the mutation may not significantly alter the electrostatic potential of the CSNK2B protein (Figure 3B). However, the stability analysis (ΔΔG) of p.Thr90Pro using DynaMut2, SAAFEC-SEQ, and DUET indicated that the mutation would lead to slight instability of the protein (ΔΔG < 0), yet with a relatively small difference (ranging from −0.3 to −0.06), suggesting that the mutation might affect protein function by disrupting local hydrogen bonds rather than inducing global unfolding (Figure 3E).

## 4 Discussion

The clinical features of this case, including early childhood-onset epilepsy and neurodevelopmental deficits, align with the POBINDS phenotypic spectrum. Whole exome sequencing identified a *de novo* missense mutation in CSNK2B (c.268A > C; p.Thr90Pro), classified as a likely pathogenic under ACMG guidelines (PM2, PM6, PP2, PP3). Computational modeling revealed localized structural perturbations, including disrupted hydrogen bonding between TYR87 and GLY93 (Figure 3E), yet predicted minimal thermodynamic destabilization (ΔΔG < 1 kcal/mol). This dichotomy highlights the challenge of interpreting in silico data for dynamic complexes like protein kinase CK2, where subtle conformational changes may impair subunit interactions without compromising global stability (2, 3). Growing evidence supports CSNK2B haploinsufficiency as the central pathogenic mechanism in POBINDS (15, 16). Truncation mutations (nonsense mutations/frameshift mutations) account for the majority of reported pathogenic alleles (17), which are likely to result in complete loss of function through nonsense-mediated decay. In contrast, hypomorphic missense variants such as p.Thr90Pro may reduce functional CK2β availability, disrupting holoenzyme stoichiometry (2, 3) and contributing to the observed phenotypic continuum (18). Structural analyses suggest the Thr90 → Pro substitution, located within the topoisomerase II interaction domain (18), introduces torsional constraints that alter local backbone flexibility (Figures 3A,D), potentially impairing CK2β's scaffolding role in DNA damage response complexes (6). The patient's mild phenotype contrasts with severe manifestations typical of truncating variants (1, 19), possibly reflecting residual CK2β activity due to the mutation's location outside catalytically critical domains (e.g., KET box-like/zinc finger motifs) (5). While missense variants generally associate with attenuated phenotypes (10, 17), exceptions exist depending on mutation location and modifier factors (16, 20), underscoring the absence of definitive genotype-phenotype correlations (17).

Epileptic seizures are the most prominent clinical feature of POBINDS, with generalized tonic-clonic and myoclonic seizures being the primary seizure types (11, 16, 17, 19–22). Other reported seizure types include absence, tonic, and tonic-clonic seizures. While the mechanism underlying these seizures remains unclear, it may involve CK2-mediated phosphorylation of calmodulin, which promotes its binding to KCNQ2 and enhances KCNQ2 channel activity (23). Eyelid myoclonia with absence, also known as Jeavons syndrome, is a genetic generalized epilepsy with childhood-onset recognized by the International League Against Epilepsy (ILAE) as a distinct epilepsy syndrome (24). It is characterized by eyelid myoclonia (with or without absence seizures), typically triggered by eye closure or photic stimulation. Although its etiology is strongly linked to genetics, a definitive causative gene has yet to be identified. Candidate genes reported in association with Jeavons syndrome include *SYNGAP1, KIA02022/NEXMIF, RORB, CHD2, GABRA1, SLC2A1, KCNB1,* and *NAA10,* which are involved in neuronal development, migration, function, and genetic regulation (25). Gokce-Samar Z et al. reported a case meeting all electroclinical criteria for epilepsy with eyelid myoclonia and absences (EMA) associated with an Xq25 microduplication spanning the entire STAG2 sequence (26). STAG2 encodes a core subunit of the cohesin complex that is involved in chromatin organization, transcriptional regulation, DNA repair, and control of downstream gene expression (27). ATP1A3, a member of the sodium-potassium ATPase gene family, has also been implicated in the pathogenesis of Jeavons syndrome (28, 29). *ATP1A3* maintains ion gradients, modulates electrophysiological activity, and participates in various signaling pathways. Mutations in *ATP1A3* may disrupt ion gradient-dependent signaling required for neuronal migration, ultimately leading to cortical laminar organization abnormalities (30, 31). Intriguingly, *CSNK2B*, the causative gene identified in this case, is involved in regulating analogous biological processes through its role in neuronal progenitor proliferation via Wnt/β-catenin signaling, apoptosis modulation through BCL2 phosphorylation, and cell migration by cytoskeletal reorganization. In this case, the patient exhibited seizures triggered by eye closure lasting approximately 2 s, with or wiathout absence seizures, along with the characteristic EEG features of 3–3.5 Hz spike-and-wave complexes. This phenotypic overlap, combined with *CSNK2B*'s established neurodevelopmental functions, positions *CSNK2B* as a novel candidate gene for Jeavons syndrome. Further studies replicating this association across independent cohorts are warranted.

Intellectual and developmental disabilities are the key features of POBINDS. Cognitive impairment is often correlated with epilepsy severity, and intellectual and developmental disabilities may persist even after seizure control. Studies have reported that over 80% of patients experience language impairment, and one-third have moderate to severe cognitive impairment (11, 21). In this case, the child's intelligence was near the borderline range with mild cognitive impairment. However, with appropriate education and consistent training, the child may develop sufficient functional abilities to achieve independent daily living.

In conclusion, we report a novel missense variant (c.268A > C; p. Thr90Pro) in the *CSNK2B* gene, which is the underlying cause of a case of Pobinds syndrome (POBINDS) characterized by Jevons syndrome. This finding expands the spectrum of mutations associated with CSNK2B-related diseases and provides preliminary evidence that the *CSNK2B* gene may be involved in the pathogenesis of eyelid myoclonus. Future studies may consider functional characterization of nerve cell models derived from children to explore genotype-phenotype correlations, particularly to determine whether missense variants located outside the catalytic domain are associated with reduced phenotypes such as Jevons syndrome.

## Data Availability

The datasets presented in this article are not readily available because of ethical and privacy restrictions. Requests to access the datasets should be directed to the corresponding authors.
