# Supplementary material for: Case Report: A case of Poirier–Bienvenu neurodevelopmental syndrome manifesting primarily as eyelid myoclonia
Source: Front Pediatr. 2025 Sep 3;13:1583346. doi: 10.3389/fped.2025.1583346 (PMC12440885; doi:10.3389/fped.2025.1583346)
Supplement: Supplementary file 3 [file Datasheet1.pdf]

If you use this service, could you please send us a mail to npsang@ibcp.fr with details about your usage of the NPSA service (tools used, frequency, type of sequence, ..) ? Could you explain what makes this service unique for you ? Could you please add information about your country and your laboratory ? Thanks

Job **SOPMA** (ID: b9492f28380c) is running on **NPS@** server (started on 20250422-090427).
Results will be shown below. **Please wait and don't go back.**

In your publication cite :
NPS@: Network Protein Sequence Analysis
TIBS 2000 March Vol. 25, No 3 [291]:147-150
Combet C., Blanchet C., Geourjon C. and Deléage G.

Running--

SOPMA result for : CSNK2B_WT

Abstract Geourjon, C. & Deléage, G., SOPMA: Significant improvement in protein secondary structure prediction by consensus prediction from multiple alignments., Cabios (1995) 11, 681-684

View SOPMA in: [AnTheProt (PC) , Download...] [HELP]

```
       10        20        30        40        50        60        70
        |         |         |         |         |         |         |
MSSSEEVSWISWFCGLRGNEFFCEVDEDYIQDKFNLTGLNEQVPHYRQALDMILDLEPDEELEDNPNQSD
cccccchhhhhhhhccttcheeeecchhhhhhhcceecccccchhhhhhhhhhhhcccccccccccchhh
LIEQAAEMLYGLIHARYILTNRGIAQMLEKYQQGDFGYCPRVYCENQPMLPIGLSDIPGEAMVKLYCPKC
hhhhhhhhhhhhhhhheeechhhhhhhhhhhhhtcccccceeecttccccccccccccccceeeeecccc
MDVYTPKSSRHHHTDGAYFGTGFPHMLFMVHPEYRPKRPANQFVPRLYGFKIHPMAYQLQLQAASNFKSP
cceeccccccccccttceecccccheeeeecttcccccccccccccceeeeeeccccchhhhhhccccccc
VKTIR
eeeee
```

```
Sequence length :   215
```

SOPMA :

Alpha helix	(Hh) :	67	is	31.16%
3_{10} helix	(Gg) :	0	is	0.00%
Pi helix	(Ii) :	0	is	0.00%
Beta bridge	(Bb) :	0	is	0.00%
Extended strand	(Ee) :	37	is	17.21%
Beta turn	(Tt) :	9	is	4.19%
Bend region	(Ss) :	0	is	0.00%
Random coil	(Cc) :	102	is	47.44%
Ambiguous states (?)	:	0	is	0.00%
Other states	:	0	is	0.00%

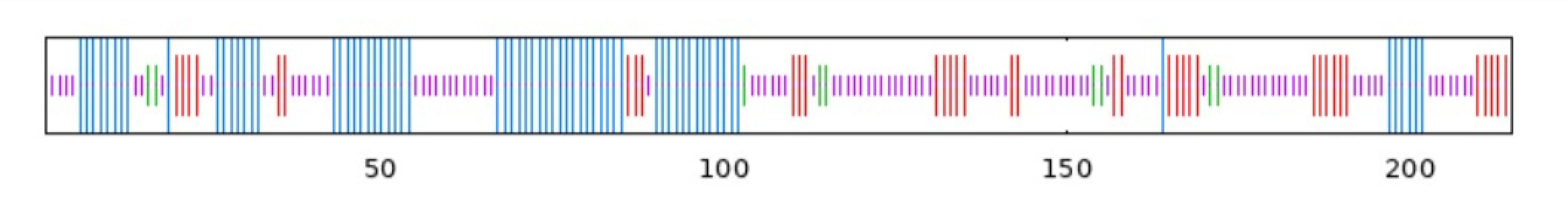

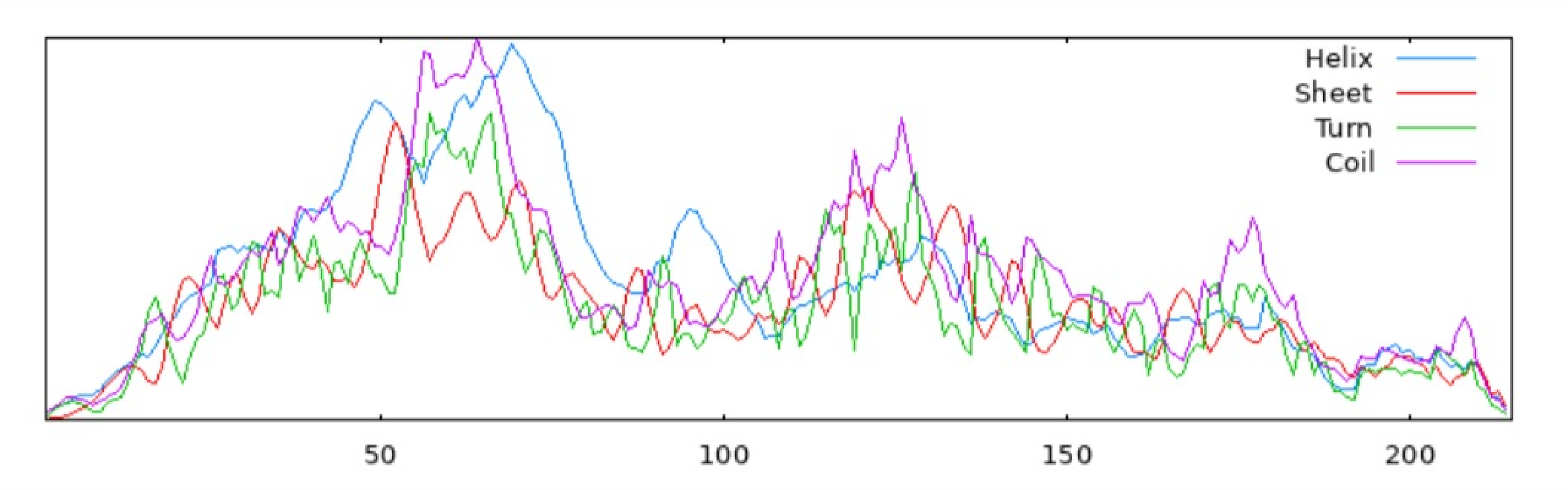


```
Parameters :
   Window width         :  17
   Similarity threshold :   8
   Number of states     :   4
```

Prediction result file (text): [SOPMA]
Intermediate result file (text): [BLASTP on NRPROT] [CLUSTALW]

*User : public **Last modification time :** Tue Apr 22 09:06:47 2025. **Current time :** Tue Apr 22 09:06:47 2025*

Copyright 2016 PBIL-IBCP-Lyon
