# Supplementary material for: Case Report: A case of Poirier–Bienvenu neurodevelopmental syndrome manifesting primarily as eyelid myoclonia
Source: Front Pediatr. 2025 Sep 3;13:1583346. doi: 10.3389/fped.2025.1583346 (PMC12440885; doi:10.3389/fped.2025.1583346)
Supplement: Supplementary file 4 [file Datasheet2.pdf]

If you use this service, could you please send us a mail to npsang@ibcp.fr with details about your usage of the NPSA service (tools used, frequency, type of sequence, ..) ? Could you explain what makes this service unique for you ? Could you please add information about your country and your laboratory ? Thanks

Job **SOPMA** (ID: 782883bab609) is running on **NPS@** server (started on 20250422-090439).
Results will be shown below. **Please wait and don't go back.**

In your publication cite :
NPS@: Network Protein Sequence Analysis
TIBS 2000 March Vol. 25, No 3 [291]:147-150
Combet C., Blanchet C., Geourjon C. and Deléage G.

Running---

SOPMA result for : CSNK2B_MUT

Abstract Geourjon, C. & Deléage, G., SOPMA: Significant improvement in protein secondary structure prediction by consensus prediction from multiple alignments., Cabios (1995) 11, 681-684

View SOPMA in: [AnTheProt (PC) , Download...] [HELP]

```
        10        20        30        40        50        60        70
         |         |         |         |         |         |         |
MSSSEEVSWISWFCGLRGNEFFCEVDEDYIQDKFNLTGLNEQVPHYRQALDMILDLEPDEELEDNPNQSD
ccccchhhhhhhhhccttcheeeecchhhhhhhcceecccccchhhhhhhhhhhhccccccccccchhhh
LIEQAAEMLYGLIHARYILPNRGIAQMLEKYQQGDFGYCPRVYCENQPMLPIGLSDIPGEAMVKLYCPKC
hhhhhhhhhhhhhhhheeechhhhhhhhhhhhhttttccceeecttcccceeecccccccceeeeecttc
MDVYTPKSSRHHHTDGAYFGTGFPHMLFMVHPEYRPKRPANQFVPRLYGFKIHPMAYQLQLQAASNFKSP
cceeccccccccccttceeccccchecccccccccccccccccceeeeeeccccccccccccccccc
VKTIR
eeeee
```

```
Sequence length :   215
```

```
SOPMA :
   Alpha helix     (Hh) :    63 is  29.30%
   3_10 helix      (Gg) :     0 is   0.00%
   Pi helix        (Ii) :     0 is   0.00%
   Beta bridge     (Bb) :     0 is   0.00%
   Extended strand (Ee) :    40 is  18.60%
   Beta turn       (Tt) :    12 is   5.58%
   Bend region     (Ss) :     0 is   0.00%
   Random coil     (Cc) :   100 is  46.51%
   Ambiguous states (?)  :     0 is   0.00%
   Other states          :     0 is   0.00%
```

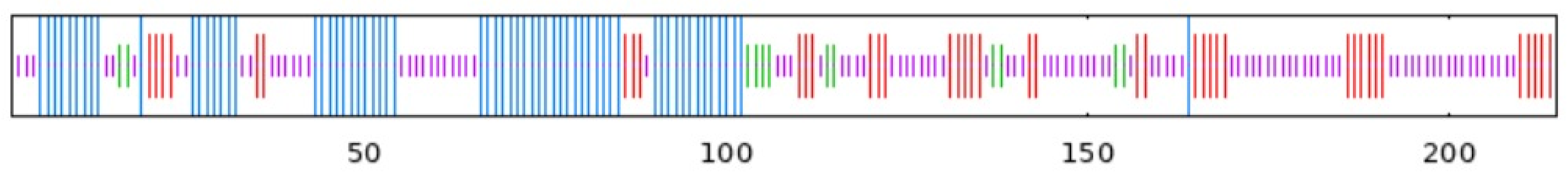

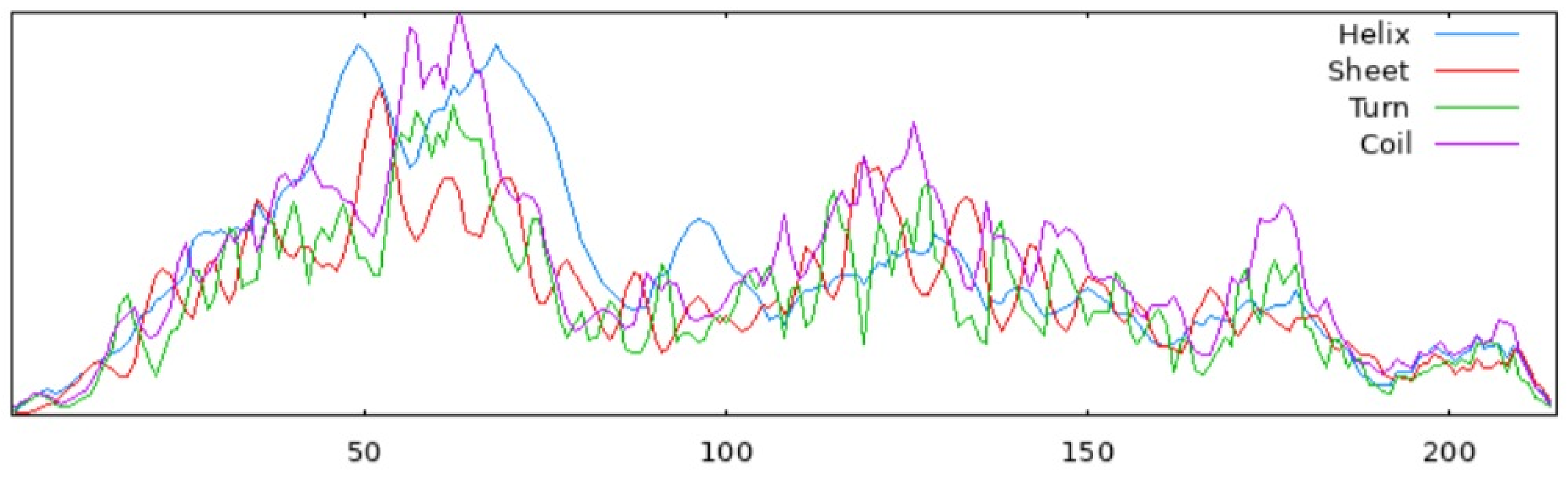


```
Parameters :
   Window width          :  17
   Similarity threshold  :   8
   Number of states      :   4
```

Prediction result file (text): [SOPMA]
Intermediate result file (text): [BLASTP on NRPROT] [CLUSTALW]

User : public ***Last modification time :*** *Tue Apr 22 09:06:46 2025.* ***Current time :*** *Tue Apr 22 09:06:46 2025*

Copyright 2016 PBIL-IBCP-Lyon
